# A longitudinal cohort study on benefit finding evolution in Chinese women breast cancer survivals

**DOI:** 10.1038/s41598-021-99809-5

**Published:** 2021-10-19

**Authors:** Weiyun Bi, Huaning Wang, Guitao Yang, Cailin Zhu

**Affiliations:** 1 Department of Thoracic Surgery, The First Affiliated Hospital, Xi’an Jiaotong University, 277 West Yanta Road, Xi’an, 710061 China; 2 Department of Psychosomatics, Xijing Hospital, The Fourth Military Medical University, 127 Changle West Road, Xi’an, 710032 China; 3 Clinical Skills Training Center, Xijing Hospital, The Fourth Military Medical University, 127 Changle West Road, Xi’an, 710032 China

**Keywords:** Psychology, Health care

## Abstract

Even though the prevalence of benefit finding (BF) has been empirically shown to exist among breast cancer (BC) survivals, how does benefit finding evolve over time remains inadequately investigated. The objective of this cohort study is to examine how BF evolves over time among Chinese breast cancer survivals and determine the demographic, medical and psychosocial factors that can sustain BF increase over time. Participants were 486 women with different stages of breast cancer (stages I, II and III) followed from completion of primary treatment. Analysis were performed on the data collected during 2014–2019. During the assessment, each participant completed self-report questionnaires of characteristics and benefit finding at six time points with the interval of 6 months since BC diagnosis. The relationships between demographic, medical and psychosocial characteristics and benefit finding evolution over time were examined using mixed models. Participants reported mixed results on the evolving patterns of benefit finding: 28% reported an upward trend in BF scoring over time, 49% instead reported an downward trend, and the remaining 23% reported no obvious change. Our study has shown that some well-known covariates of benefit finding, e.g. education, income, and social support, are not associated with BF trends. In comparison, levels of spirituality and disease coping at diagnosis can more reliably predict BF evolution over time. Identifying the sustaining factors of benefit finding in the experience of breast cancer is the key to design effective psycho clinical solutions for patients’ long-term post-traumatic growth. As time goes by, breast cancer patients may experience less benefit finding. Our results strongly indicate that benefit finding can be sustained and increased by encouraging attempts at meaning-making and active disease coping during breast cancer treatment. To the best of our knowledge, this study is among the first to examine the evolution trends of benefit finding over time on breast cancer survivals and determine their psychosocial predictors in developing countries.

## Introduction

Among women, breast cancer (BC) is one of the most common malignant diseases worldwide^1^. As prognosis of BC has improved over the last years, research on quality of life of BC survivors has become increasingly important. Previous studies have shown that many BC survivors reported positive changes in their experience of disease (e.g. improved relationships, deeper appreciation for life and reorganization of life’s priorities)^2,3^. Benefit finding (BF) has been proposed as one of two major constructs of positive consequences of cancer, which also include post-traumatic growth (PTG)^4–6^. BF was defined as the process in which a patient re-assigns positive value to his (or her) illness based on the benefits he (or she) identifies. In contrast, PTG refers to the more general positive changes in life perspective, interpersonal relationship and self-perception. While BF is supposed to start immediately after diagnosis, PTG, referring to the active change in one’s capacity to deal with adverse events, may develop even years after diagnosis^7–9^. Benefit finding has been frequently examined in the context of breast cancer survivorship. Early investigations of BC benefit finding have demonstrated that most women reported positive life changes due to their diagnosis^10^. To date, the existing studies on BC benefit finding mainly investigated the covariates of benefit finding in various demographic groups^11–14^. They showed that greater benefit finding may be associated with miscellaneous factors, e.g. education level, spirituality, illness intrusiveness, social support and mental health. However, even though benefit finding is typically assumed to increase over time following cancer diagnosis^15^, as increased time allows for additional cognitive processing of the diagnosis, preliminary findings in the literature are actually mixed. Some studies reported an increase in benefit finding over time after cancer diagnosis^5^, while others instead reported a decrease^16,17^. It is noteworthy that the primary objective of these studies was to identify the covariates of benefit finding. The issue of BF evolution over time unfortunately remains inadequately investigated.

However, the issue of how BF evolves over time in breast cancer survivals is critically important given that BF and PTG are closely correlated^18,19^. In other words, increasing BF after diagnosis can effectively boost longer-term PTG of cancer survivals, thus improve their quality of life. The aim of the present study is twofold: (a) to examine the trends of BF evolution over time among breast cancer survivals; and (b) to identify the factors that can sustain BF increase over time. We hypothesize that BC survivors would generally report some extent of benefit finding after diagnosis, but whether BF can be sustained over time and develop into longer-term PTG depends on some potential psycho and social factors, which include age, education level, income, social support, spirituality and active disease coping.

## Methods

### Participants

The cohort study was conducted with 486 women aged between 20 and 50 years and diagnosed with stage I–III breast cancer between 2014 and 2017. Women were recruited through the first affiliated hospital of the Air Force Military Medical University and screened by chart review. Our study did not involve women aged above 50 because most of patients in the hospital were aged below 50, and more importantly, most of the patients aged above 50 did not consent to participate. We also excluded those with severe chronic diseases, e.g. coronary heart disease, chronic obstructive pulmonary disease (COPD) and chronic renal failure.

Of 1028 eligible women, 667 consented to participate. The two most cited reasons for refusing to participate are: (1) do not want to reveal personal experience, even for scientific study; (2) do not want to be bothered. Those consenting to participate were required to sign a formal consent form. Each participant was then emailed questionnaires to complete and return response. For each participant, the first/baseline questionnaire was sent out within 6 months of diagnosis and supposed to be completed within two weeks thereafter. The follow-up questionnaires were sent 6, 12, 18, 24, and 30 months after the baseline time point respectively. Similarly, they were also supposed to be completed within 2 weeks thereafter. Because the baseline questionnaires were administered to participants at differing lengths of time following diagnosis, we used the dates of completion of each survey along with the date of diagnosis to create a continuous variable of time since diagnosis (in months).

In appreciation of their time, enrolled participants could select from several promotional items (< 100 RMB value). Due to cancer recurrence or lost contact (moving out of the city of Xi’an), some of the initial participants dropped out during our study. During the assessment period, 637 participants completed the 1st-year assessment, 579 completed the first 2-year assessment, and 486 completed the entire 3-year assessment. Given our focus on benefit finding over time, we limited analysis to the 486 participants who had completed the entire 3-year assessment and were still disease free at the end of assessment.

### Measures

We focus on the demographic, medical and psychosocial characteristics, which may to some extent affect benefit finding.

**Table 1:** Characteristics of the study sample (N = 486) and BF scoring at baseline.

Demographic/medical characteristics	N (%)	BFS-baseline M (SD)
**Age (years)**		
< 30	85 (17)	57.35 (4.22)
$\ge$30, < 40	284 (59)	55.34 (3.14)
$\ge$40, < 50	117 (24)	57.45 (4.38)
**Marital status**		
Married	414 (85)	56.39 (2.96)
Others	72 (15)	55.10 (5.68)
**Education**		
$\le$ High school	223 (46)	54.26 (3.44)
College	187 (38)	57.38 (3.60)
> College	76 (16)	58.98 (4.32)
**Income (CNY monthly)**		
$\le$ 3000 (450 USD/400 EUR)	166 (34)	54.18 (2.78)
> 3000, $\le$ 6000 (900 USD/800 EUR)	213 (44)	55.96 (4.33)
> 6000	107 (22)	59.81 (3.67)
**Employment status**		
Fully employed	420 (86)	56.28 (3.68)
Other	66 (14)	55.69 (5.86)
**Cancer stage**		
I	238 (49)	56.76 (3.13)
II	167 (34)	55.48 (4.21)
III	81 (17)	56.03 (4.38)
**Surgery**		
Lumpectomy only	287 (59)	56.16 (4.24)
Mastectomy only	126 (26)	55.67 (3.34)
Mastectomy/reconstruction	73 (15)	58.99 (6.78)
**Radiation therapy**		
Yes	397 (82)	56.35 (3.48)
No	89 (18)	55.53 (4.28)
**Chemotherapy**		
Yes	338 (70)	56.27 (3.86)
No	148 (30)	56.04 (4.11)

#### Demographic characteristics

Each participant needs to report their age, marital status, educational level, income, and employment status. We classified the ages into three groups: under 30, between 30 and 40, and finally above 40, which receive the variable values of 5, 10 and 15 respectively. Marital status was defined to be married (value = 10) or others (value = 5), which include divorced and unmarried. Education levels included high school, college and postgraduate, which receive the values of 5, 10 and 15 respectively. Monthly income also included three levels, under 3000 CNY (or 450 USD/400 EUR), between 3000 CNY and 6000 CNY (or 900 USD/800 EUR), and above 6000 CNY, which receives the values of 5, 10 and 15 respectively. Employment status was defined to be fully employed (value = 10) or others (value = 5), which include unemployed or part-time.

#### Medical factors

A medical chart review was performed upon completion of primary treatment. With the help of five nurses at the Department of Oncology, our research team collected the relevant medical information of participants from their medical records. The collected information included cancer stage (I or II or III), type of surgery, receipt of radiation, and receipt of chemotherapy. For statistical analysis, cancer stage I receives the value of 5, stage II the value of 10, and stage III the value of 15. On type of surgery, surgery of lumpectomy receives the value of 5, surgery of mastectomy the value of 10, and surgery of mastectomy/reconstruction the value of 15. It is noteworthy that SNB and Axillary dissection may be performed with surgery; however, our study did not make further differentiation. On both receipt of radiation and receipt of radiation, the *no* answer receives the value of 5 while the *yes* answer receives the value of 10.

#### Social support

The RAND Social Support Scale measured participants’ evaluation of the resources provided by their social network^20^. It measures four aspects of support: emotional, tangible, affection, and social interaction^21^. A total score sums the four categories with a possible total score ranging from 20 to 100 (higher scores means greater support).

#### Spirituality

We assessed Spirituality by the Functional Assessment of Chronic Illness Therapy—Spiritual Well-Being (FACIT-Sp) scale^22,23^. The metric of FACIT-Sp was developed with the input of cancer patients, psychotherapists, and religious/spiritual experts (e.g., hospital chaplains), who were asked to describe the aspects of spirituality and/or faith that contributed to quality of life. The responses emphasized a sense of meaning in life, harmony, peacefulness, and a sense of strength and comfort from one’s faith. This 12-item scale contains two subscales: meaning/peace and faith. Since the participants in our study are mostly Han Chinese, who are usually not religiosity-informed, we mainly measured spirituality scoring on the meaning/peace subscale. The eight-item meaning/peace subscale assesses a sense of meaning, peace, and purpose in life (Cronbach’s $\alpha$ = 0.83). Higher scores indicate greater spiritual well-being.

#### Disease coping

We assessed disease coping by the Brief COPE^24^. The subscales from the Brief COPE include active coping, planning, acceptance, instrumental and emotional social support seeking (2 items each). Items were completed in reference to “what you have been doing to cope with your experience of cancer, including your current physical or emotional concerns related to your cancer experience” and were rated on 4-point scale (1 = I don’t do this at all; 4 = I do this a lot). The means of each subscale were used to compute an overall composite mean, giving each subscale equal weight in the composite. Not surprisingly, subscales were positively and significantly correlated ($\hbox {r}=0.15$ to 0.71, $\hbox {p}<0.05$). It is noteworthy that internal consistency was acceptable in the current study ($\alpha$ = 0.79).

#### Benefit finding

We measured the Benefit Finding Scale (BFS) by a Chinese version^25^, which was translated from the original BFS proposal^10^. The Chinese version consists of totally 19 items and asks participants to respond to “Have you found any positive change from the experience of your cancer” on a scale from 1 (not at all) to 4 (a great deal of change). Possible BF scores vary from 19 to 76. In our study, Cronbach’s $\alpha$ for the BFS was 0.86.

### Analytical strategies

All analyses were conducted using IBM SPSS Statistics 25. Multivariate linear models were used to assess effects of demographic, medical, and psychosocial variables on BFS scores at baseline, and random coefficient models were used to assess effects of these variables on BFS scores in longitudinal analysis over all time points. The null hypothesis is that BF scores are independent of the test variables. With the significance level set at 0.05 ($\alpha =0.05$), a variable is therefore considered to be correlated with BFS if its *p*-value is less than 0.05. With more than 1000 observations, our post-hoc sensitivity analyses demonstrated at least 80% power to detect a medium-sized effect of $d = 0.3$ at $\alpha = 0.05$ with an intra-class correlation of 0.582.

For trend analysis, time was calculated as multiples of 6-months since diagnosis and included in the model using linear term. For each participant, we estimate its BF trending slope, which is denoted by $\beta$, by a linear regression model. We categorized BFS trend patterns into three trends (upward, downward or no obvious change) according to their estimated $\beta$ values. Specifically, $\beta \ge 0.3$ means upward, $\beta \le -\, 0.3$ means downward, while $- \,0.3<\beta < 0.3$ means no obvious change. Then, we analyzed the association between various variables and BF trending slopes (or $\beta$) by multivariate linear models. The null hypothesis is that BF trending slopes are independent of the test variables. With the significance level set at 0.05 ($\alpha =0.05$), a variable is therefore considered to be a predictor of BF trends if its *p*-value is less than 0.05.

### Ethics approval and consent to participate

The experimental protocol was established, according to the ethical guidelines of the Helsinki Declaration and was approved by the Research Ethics Committee of Xijing Hospital, the Air Force Military Medical University. Written informed consent was obtained from individual or guardian participants.

## Results

### Covariates of BF

The detailed characteristics of the sample have been presented in Table 1. It is clear that benefit finding was found on the participants after cancer diagnosis. The results of association analysis have been presented in Table 2. It can be observed that at baseline, BF score was significantly associated education level, income level, social support, spirituality, and disease coping ($p\le 0.05$). It was however not observed to be related to any other studied social and psycho variable ($p>0.05$).

**Table 2:** Association of demographic, clinical, and psychosocial characteristics with Benefit Finding scores.

Characteristics	Baseline model		Longitudinal model^a^	
	$\beta$ (SE)	p-value	$\beta$ (SE)	p-value
Age at Dx (per decade)	− 1.35 (1.64)	0.2622	− 1.92 (0.89)	0.2212
Education	1.68 (0.56)	0.0385*	1.79 (0.76)	0.0281*
Income (RMB/month)	1.42 (0.38)	0.0475*	2.04 (0.49)	0.0388*
Stage	− 0.66 (0.31)	0.2675	− 0.55 (0.28)	0.3672
Surgery	0.84 (0.21)	0.1035	1.94 (0.65)	0.1286
Chemotherapy	− 0.34 (0.11)	0.0943	− 0.53 (0.15)	0.1076
**Social support**				
Baseline	1.98 (1.30)	0.0021*	2.19 (1.34)	0.0009*
Change from baseline			1.94 (0.39)	0.0003*
**Spirituality (meaning and peace)**				
Baseline	1.32 (0.51)	0.0004*	2.46 (0.57)	0.0002*
Change from baseline			1.28 (0.18)	0.0001*
**Disease coping**				
Baseline	3.36 (2.62)	0.0001*	3.57 (1.30)	< 0.0001*
Change from baseline			3.26 (0.43)	< 0.0001*

Demographic and medical variables that were nonsignificant in the models (marital status, employment status and radiation therapy) are not shown.

^a^Longitudinal model using baseline value and change from baseline for time varying covariates.

*$$p\le 0.05.$$

The results of longitudinal association analysis have also been shown in Table 2. The results indicated that education, income, social support, spirituality and disease coping are closely correlated with time-varying benefit finding scoring. It can be observed that both social support and use of active coping strategies was highly correlated with changes of BFS over time. A 1-point increase in time-varying social support means a 1.94-point increase on BF scoring, while a 1-point increase in disease coping predicted a 3.26-point increase. Education level and spirituality are also correlated with BFS changes, but to a lesser extent.

### Predictors of BFS trends

The results of BFS evolution analysis are presented in Table 3. It can be observed that the overall BFS of the sample decreases over time. In many participants, the first BF scoring after diagnosis (the baseline value) is the highest and it then generally decrease over time. Among all the participants, only 28% reported an upward trend over time ($\beta \ge 0.3$), while around half (49%) instead reported a downward trend ($\beta \le -\, 0.3$); the remaining 23% reported no obvious change.

**Table 3:** Categorization of BFS trend patterns (N = 486).

Characteristics	N (%)	Slope (M)	SD
Entire population	486 (100)	− 0.133	0.442
$\beta \ge 0.3$	136 (28)	0.391	0.081
$-\,0.3<\beta <0.3$	112 (23)	− 0.083	0.165
$\beta \le -\,0.3$	238 (49)	− 0.479	0.133

**Table 4:** Predictors of BFS trend patterns by multivariate linear model (N = 486).

Characteristics	$\beta$ (SE)	p-value
Age at Dx (per decade)	− 0.024 (0.011)	0.2310
Education	0.045 (0.024)	0.1004
Income	0.056 (0.013)	0.2684
Cancer stage	− 0.048 (0.011)	0.2122
Surgery	0.084 (0.036)	0.1367
Chemotherapy	− 0.028 (0.010)	0.1235
Social support	− 0.032 (0.017)	0.1068
Spirituality	0.114 (0.068)	0.0289*
Disease coping	0.256 (0.126)	0.0004*

*$$p \le 0.05.$$

The results on association between the characteristics of the participants and BFS trends are presented in Table 4. It can be observed that higher levels of spirituality and disease coping at baseline strongly indicated an increasing BFS trend ($p\le 0.05$). Specifically, among the participants with spirituality scoring above 80-points, the average slope of BFS trending is 0.266, $\beta =0.266$, which is much higher than the average slope of the entire sample ($\beta = -\, 0.133$). The results w.r.t disease coping are similar. Among the participants with coping scoring above 80-points, the average slope coefficent of BFS trending is 0.438, considerably higher than the average slope of the entire sample.

It is worthy to point out that some covariates of BFS such as education, income and social support are not reliable predictors of BFS trending pattern. In particular, higher social support scoring at baseline did not not necessarily predict an upward BFS trend. Among the participants with the baseline social support above 80-point, the average slope of BFS trending is only − 0.113, which indicates no obvious change in BF scoring. Higher education and income similarly did not indicate an upward trend either. Among the participants with at least Bachelor’s degree, the average slope is only − 0.102. Among the participants with the monthly income above 6000 CNY, which can be considered high-salary earners in western China, the value is similarly at − 0.096. None of them indicated an upward trend.

## Discussion

Despite many negative physical and quality of life sequelae following breast cancer diagnosis and treatment, many patients reported positive changes in their lives following cancer diagnosis^3,26^. Even though there exist many studies focused on identifying the covariates of benefit finding among various demographic groups^11–14^, the issue of how benefit finding evolves over time has not been adequately investigated. To our knowledge, this study is among the first to examine the trends of benefit finding evolution over time and their predictors in developing countries.

Consistent with previous studies conducted in American and European clinics^27,28^, our sample also reported considerable benefit findings among the participants after cancer. In the longitudinal association analysis, similar to what were observed in previous studies^3,14,29,30^, social support, spirituality and disease coping are closely correlated with changes of benefit finding scoring over time. It is noteworthy that education and income levels are associated with BF scoring as well. One probable explanation for this observation is that the patients with higher education and income levels have relatively richer social activities, thus receive more social support. Consistent with the Chinese culture that emphasizes kinship, our study has shown that perceived support from family was greater than support from other sources among Chinese BC survivors. Furthermore, both support from society and support from friends have a stronger association with BF scoring among younger survivors than among older survivors.

Interestingly, the results of trending model indicated that the average BFS in our sample actually decreased a little bit over time since diagnosis, at a rate of − 0.133 per 6-month. Although it makes conceptual sense that benefit finding would increase over time following cancer diagnosis, our results indicated the opposite, which has also been reported in other demographic groups^14^. There are several probable explanations for this effect. First, the overall high levels of benefit finding at the time of cancer diagnosis may have resulted in a “ceiling effect” and regression to the mean over time. More importantly, the income levels of most patients in our sample were relatively low ($< 6000$ CNY per month). As time since diagnosis increases, survivors may struggle for bread and butter while cancer becomes a less salient stressor.

Most importantly, as shown in Table 4, spirituality and disease coping at diagnosis were positively related to benefit finding evolution over time. It can be observed that even though Han Chinese are mostly non-religious, life meaning and active coping are still critically important for sustaining benefit finding over time. Our results strongly indicated that regardless of demographics, purpose-oriented or problem-solving rumination generally leads to higher levels of benefit finding, while aimless and passive way of life usually leads to lower levels. It is interesting to observe that the covariates of benefit findings may not reliably indicate its trending pattern. Specifically, the covariates of benefit finding observed in association analysis including education, income and social support can not accurately predict BFS trends. Even though higher education and income usually lead to higher initial levels of benefit finding, unfortunately they can not sustain its increase over time. Our data actually indicated the opposite: with all the participants with the income level above 6000 CNY per month, the score of benefit finding decreases a little bit after the baseline measured at diagnosis. Among the participants with at least a Bachelor’s degree, the level of benefit finding shows no obvious change after the baseline. In terms of social support, even though it is strongly correlated with benefit finding at almost all time points, higher level of social support at baseline may not sustain itself over time, thus does not necessarily indicate an upward BFS trend.

## Conclusion

Some important results emerge from our study. First, benefit finding usually develops very soon following a breast cancer diagnosis. However, it does not necessarily increase over time. On one hand, benefit finding may be supported by the salient threat of cancer recurrence, as threat and distress appear to be important aspects in the development of BF. But for patients in less developed areas with limited income, the threat may become a less salient stressor for survivors over time. Secondly, our study showed that even though benefit findings may decrease over time, the efforts of meaning making and active coping can effectively counter its negative trend. Uncovering the underlying factors sustaining positive BFS changes in the experience of cancer is the first step to increase long-term post-traumatic growth of breast cancer patients.

Our study implies that psychosocial BC interventions should focus on the approaches that support personal spiritual growth. There exist some examples of this intervention in the literature. For example, it has been found that a spiritually-based educational intervention for prostate cancer screening increased knowledge and self-efficacy for screening among Black men^29^. Such studies provided preliminary evidence for the potential benefit of spirituality-informed treatments in behavioral oncology. In addition, in the Chinese culture emphasizing kinship, collaborating with family-support organizations is one of potential opportunities to encourage active coping in BC survivors.

However, this study had several limitations, which need to be further investigated in future work. First, the sample has limited diversity: (1) participants consist of mostly non-religious Han Chinese women, which may limit generalizability; (2) due to limited sample size, our study did not involve the BC patients aged above 50. Future studies should consider recruiting a larger sample to replicate these findings in a more diverse Chinese or Asian population. Secondly, our study focused on the benefit finding immediately after diagnosis. Predictors of BFS trends were estimated based on variable measurements at baseline. The psychosocial characteristics of cancer patients may vary over time. Therefore, it may be necessary to increase the assessment window and investigate how long-term post-traumatic growth evolves long after diagnosis.

## Data Availability

The datasets generated during and/or analysed during the current study are not publicly available due to the privacy policy concerning private information of active army personnel, but are available from the corresponding author on reasonable request.
